# Nutritional Composition and Sensory Acceptability of Stinging Nettle (*Urtica simensis*) Flour-Supplemented Unleavened Maize (*Zea mays L.*) Flatbread (*Kitta*)

**DOI:** 10.1155/2021/6666358

**Published:** 2021-08-14

**Authors:** Tona Zema Diddana, Gezahegn Nigusse Kelkay, Endale Elisho Tescha

**Affiliations:** School of Nutrition, Food Science and Technology, Hawassa University, Hawassa, Ethiopia

## Abstract

In Ethiopia, a few studies had been conducted to improve the nutritional values and sensory acceptability of maize-based flatbread. These studies did not address indigenous edible wild green vegetables like stinging nettle (*Urtica simensis*). Consequently, there was a scientific report gap on the effect of incorporating stinging nettle leaf flour into local staple foods like flatbread. Therefore, this study was intended to investigate the nutritional composition and sensory acceptability of unleavened maize (*Zea mays L.*) flatbread (*Kitta*) supplemented with stinging nettle (*Urtica simensis*) flour. The flatbread was developed from composite flour of germinated maize and nettle leaf in a ratio of 90 : 10, 85 : 15, 80 : 20, and 75 : 25, respectively. Hundred percent (100%) nongerminated maize flour flatbread was used as control. Proximate composition, minerals (Fe, Zn, and Ca), and vitamin C contents were analyzed. The sensory acceptability test was rated by a nine-point hedonic scale. The result revealed that crude protein and fat decreased from 11.02 g to 7.21 g and 1.12 g to 0.48 g, respectively, when the amount of nettle flour supplementation increased from 0% to 25%. On the contrary, total ash, crude fiber, and total carbohydrate slightly increased from 1.84 to 3.81 g, 2.19 to 3.05, and 75.53 to 80.05 g, respectively. The calcium, zinc, and iron content significantly (*p* < 0.05) increased from 60.51 to 283.74 mg, 5.09 to 9.24 mg, and 1.72 to 3.59 mg when the amount of nettle flour increased from 0% to 25%, respectively. All sensory acceptability tests showed decrement with increasing the amount of nettle flour, but the control group has the highest acceptability.

## 1. Introduction

Unleavened flatbread, *Kitta* in local language (Amharic), is a baked product made from cereal flour [1]. It is made without adding baking powder and yeast. Flour and water are the two basic ingredients used to prepare unleavened flatbread. The flour comes from various types of cereals such as maize, wheat, rye, barley, teff, or their mixtures. Unleavened flatbread is consumed throughout the world although the recipe varies. In Ethiopia, it is instant bread usually prepared for immediate consumption or as a substitute of Injera. As a result, it is most commonly consumed traditional food in almost all rural communities and rarely by urban peoples [2].

A few studies had been conducted in Ethiopia to improve nutritional values and sensory acceptability of maize-based flatbread. For instance, Serka et al. [3] investigated the nutritional value and sensory acceptability of flatbread prepared from kocho, broad bean, and quality maize protein. Another study by Teferra et al. [4] also investigated the nutritional and sensory properties of flatbread from maize (Zea mays L.) and orange-fleshed sweet potato (Ipomoea batatas L.) flour. However, these studies done in Ethiopia were addressed indigenous edible wild green vegetables [5], such as stinging nettle (*Urtica simensis*). Some studies conducted outside Ethiopia, like Maietti et al. [6], investigated chemical composition and antioxidant performance in stinging nettle-supplemented breads. Similarly, Durovic et al. [7] also studied the effect of stinging nettle leaves and its extract enrichment of leavened bread. These studies focused on leavened bread with the addition of yeast. Moreover, these studies used white wheat as a recipe.

Stinging nettle (*Urtica simensis*) is an annual and perennial undervalued wild green herb distinguished by stinging hairs [8]. It is categorized under the genus *Urtica* of the family *Urticaceae* [1]. It mostly grows in temperate and tropical wasteland areas around the world. In Ethiopia, it grows in the highlands of the Amhara region around North and South Gondar, North and South Wollo, North Shewa, and Wag Hamra, Gojam; Oromia region around Kofole of Arsi zone and in most highlands of Sidama Zone in the southern region [9].

Stinging nettle (*Urtica* simensis) leaves are eaten in many parts of the world as cooked leaves at times of famine [5, 10, 11]. The leaves are popular especially in poor countries and lower socioeconomic classes [12]. It could be utilized as spinach like a cooked vegetable in the human diet [5, 10, 13, 14]. For instance, the leaf of nettle is used as a wild source of vegetables in rural areas of South Africa [13, 14], and some highland areas of Ethiopia [11, 15]. Nettles may also be used as juice, tea, and an ingredient in many dishes [16]. This plant is endemic to Ethiopia and identified as one of the most commonly utilized wild edible plant species in some parts of the country [17]. In Ethiopia, the young nettle leaves mixing with barley powder are utilized as side dishes such as soup and served with Injera and cooked during fasting and famine or food shortage season [18].

Stinging nettle (*Urtica simensis*) leaves add not only variety to the menu but also valuable sources of macro- and micronutrients and other bioactive compounds. Hughes et al. [19] reported that the essential amino acids in nettle are comparable to beans (*Phaseolus vulgaris*) and chicken (*Gallus gallus*). Amino acids from dehydrated nettle meal are also nutritionally better compared to alfalfa meal [9]. Another study revealed that nettle leaf powder contained higher protein compared to wheat and barley flours [20]. The fresh nettle leaves are rich in Ca and iron [18, 21], zinc [22], previtamin A [23], protein and vitamin A [18, 23], and ascorbic acid (vitamin C) [18, 24].

Utilizing such wild edible plants may help to close food gaps during famine and combat food insecurity concomitantly improving malnutrition in low-income countries. However, there is a limited scientific report on the effect of incorporating nettle leaves on the chemical composition and sensory acceptability of unleavened flatbread in Ethiopia. On the other hand, this plant is considered as poor man's food [18], weeds due to their rapid growth and soil coverage [16], and undervalued plant. Considering this scientific report gap and valuable nutritional and nutrient contribution of the nettle, this study was intended to investigate the nutritional composition and sensory acceptability of unleavened flatbread (*Kitta*) developed from stinging nettle (*Urtica simensis*) leaf and maize (*Zea mays* L.) flour.

## 2. Materials and Methods

### 2.1. Sample Preparation

Maize (*Zea mays L.*) and stinging nettle (*Urtica simensis*) were procured from Hawassa town open market and Wondogenet district, respectively. Maize was sorted, cleaned, and then soaked for 24 hrs in clean water. The soaked maize was rinsed with clean tap water. Then, it was allowed to germinate for 48 hrs and sun-dried. The young shoot of the *nettle* (*Uritca simensis*) leaf was separated from its steam using sanitized protective gloves. The leaves were washed by deionized water and then dried at room temperature in the food processing laboratory of the Department of Food Science and Technology Hawassa University, Ethiopia. Finally, the dried control maize, germinated maize (treatment recipes), and young shoot of nettle leaves were separately milled into flour passing a 1 mm diameter sieve using a laboratory miller (Thomas Wiley Mill Model 4, USA). The sieved flour was packed in a polyethylene bag and stored at room temperature until flatbread preparation.

### 2.2. Blending Ratio and Flatbread Preparation

Five treatments (SN0, SN10, SN15, SN20, and SN25) were formulated from nongerminated traditional maize flour (control), germinated maize flour, and nettle leaf flour as depicted in Table 1. The dough of the control group and treatment group was prepared by mixing the blended flour and water. The dough was manually kneaded until it became soft. The softened and smooth dough was wrapped by *enset* leaves (*Enset ventricosum*). Then, it was baked by a preheated local clay griddle called “*Mitad*” for 15-20 minutes. The baked flatbread was milled into flour. Finally, the flour was packed in a polyethylene bag and kept in desiccators until compositional analysis.

### 2.3. Nutritional Composition Analysis

The chemical composition of the flatbread was analyzed in the Ethiopian Public Health Institute (EPHI) food analysis laboratory, Addis Ababa. Proximate compositions (crude protein, moisture, crude fat, crude fiber, and total ash), vitamin C, and minerals (calcium, zinc, and iron) were analyzed by the methods of the Association of Official Analytical Chemists (AOAC) [25]. Vitamin C was analyzed by UV spectrophotometer Shimadzu (Model: UV-1800).

### 2.4. Determination of Moisture Content

Moisture content was determined by drying the samples in a drying oven (Memmert, Din 40050, West Germany). Empty dishes and lids (made of porcelain) were dried at 100°C for 1 hr. The dried dishes were then transferred to the desiccators which contain granular silica gel and cooled for 30 minutes. The dried empty dishes were weighed (**W**_1_). About 3.00 g of fresh sample were thoroughly mixed and transferred to the dried and weighed dishes. Then, dishes and their contents were weighed (**W**_2_). The contents were placed in the drying oven and dried for 3 hr at 105°C. Finally, the dishes and their contents were cooled in a desecrator to room temperature and reweighed (**W**_3_). The moisture contents of the sample were determined and expressed as follows:

(1) $$\begin{array}{c} \text{Moisture }\left(\%\right)=\frac{\left(W_{2}-W_{1}\right)*100}{W_{2}-W_{1}}, \end{array}$$

where *W*_1_ is the weight of empty dish (g), *W*_2_ is the weight of empty dish + fresh sample before drying (g), *W*_2_–*W*_1_ is the weight of sample (g), *W*_3_ is the weight of container + sample after drying (g), and *W*_2_–*W*_3_ is the loss of weight (g).

### 2.5. Determination of Crude Protein Content

Crude protein was analyzed by the Kjeldahl method using the Kjeldahl analyzer in three steps (digestion, titration, and distillation). Digestion, step 1: initially, the sample was homogenized and then 2.00 g of fresh samples was taken in a Tecator tube. Concentrated orthophosphoric acid and concentrated sulfuric acid in ratio 5 : 100, respectively, were added and mixed thoroughly. A 3.5 ml of 30% hydrogen peroxide was added step by step. Immediately after the violent reaction had ended, the tubes were shaken and placed back to the rack. Three grams of catalyst, a mixture of ground 0.5 g of selenium metal with 100 g of potassium sulfate was added into each tube and allowed to stand for about 10 minutes before digestion. Then, the tubes were lowered into protein digester when the temperature of the digester attained 370°C. The digestion was continued for about 1 hour until a clear solution was obtained. The tubes in the rack were cooled in a fume hood; 15 ml of deionized water was added and shaken to avoid precipitation of sulfate in the solution. Distillation, step 2: the digested and diluted sample solution was distilled using boric acid. Titration, step 3: the distillate was titrated using 0.1 N sulfuric acid to reddish color using the Kjeldehal apparatus (Kjeltec 2300 Analyzer unit, Foss Tecator AB, Hoganas, Sweden).

### 2.6. Determination of Crude Fat Content

The crude fat content was determined by exhaustively extracting a measured and known weight of sample in diethyl ether (boiling point 55°C) in a soxhlet extractor (model: 2055 Soxtec Foss Tecator). The ether was evaporated from the extraction flask. The amount of fat was quantified gravimetrically and calculated from the difference in weight of extraction flask before and after extraction as percentage. The extraction flasks were cleaned, dried in a drying oven (Memmert, Germany) at 70°C for 1 hour, cooled in desiccators (with granular silica gel) for 30 minutes, and then weighed (**W**_1_). The bottom of the extraction thimble was covered with about 2 cm layer of fat-free cotton. Two grams (*W*_o_) of fresh sample was added into the extraction thimbles and then covered with about 2 cm layer of fat-free cotton. The thimbles with the sample content were placed into a Soxhlet extraction chamber. The cooling water was switched on, and a 50 ml of diethyl ether was added to the extraction flask through the condenser. The extraction was conducted for about 3 hours. The extraction flasks with their content were removed from the extraction chamber and placed in the drying oven at 70°C for about one hour, cooled to room temperature in the desiccators for half an hour. The content was reweighed (*W*_2_) immediately after it was taken out of desiccators. The amount of crude fat in the sample was calculated by the difference in weight of the extraction flask before and after extraction shown as follows:

(2) $$\begin{array}{c} \text{Fat}\left(\%\right)=\frac{\left(W_{2}-W_{1}\right)*100}{W_{\text{o}}}, \end{array}$$

where *W*_o_ is the weight of fresh sample, *W*_1_ is the weight of dried flask, and *W*_2_ is the weight of extraction flask after extraction (weight of flask and fat).

### 2.7. Determination of Crude Fiber Content

The crude fiber was determined in four steps (digestion, filtration, washing, and drying and combustion) using a muffle furnace (Model: Carbolite, England). Digestion, step 1: 2.00 g (*W*_1_) of fresh sample was placed into a 600 ml beaker, and 200 ml of 1.25% H_2_SO_4_ was added. The content was then gently boiled for 30 minutes placing a watch glass over the mouth of the beaker. After 30 minutes of boiling, 20 ml of 28% KOH was added and boiled gently for further 30 minutes with occasional stirring. Filtration, step 2: the bottom of sintered glass crucible was covered with a 10 mm sand layer, and the layer of the sand was wetted with a little distilled water. The solution was poured from the beaker into sintered glass crucible, and then, the vacuum pump was turned on. The wall of the beaker was rinsed with hot distilled water several times; washings were transferred to a crucible and filtered. Washing, step 3: the residue in the crucible was washed with hot distilled water and filtered. The residue was washed with 1% H_2_SO_4_ and filtered and then washed with hot distilled water and filtered. Again, the residue was washed with 1% NaOH and filtered; again, it was washed with hot distilled water and filtered. Again, the residue was washed with 1% H_2_SO_4_ and filtered; again, it was washed twice more with hot, distilled water and filtered. Finally, it was washed with water-free acetone. Drying and combustion, step 4: the crucible with its content was dried for 2 hours in an electric drying oven at 130°C and cooled for 30 min in the desiccators (with granular silica gel) and then weighed (*W*_2_). The crucible was transferred to a muffle furnace (Gallenkamp, model FSL 340-0100, UK) and incinerated for 30 min at 550°C for ashing. The crucible was cooled in the desiccators and weighed (*W*_3_). Then, the crude fiber was calculated as a residue after subtraction of the ash shown in the following formula:

(3) $$\begin{array}{c} \text{Crude fiber}\left(\%\right)=\frac{\left(W_{2}-W_{1}\right)*100}{W}, \end{array}$$

where *W*_1_ is the weight of crucible, sand, and residue; *W*_2_ is the weight of crucible and sand; and *W* is the sample weight.

### 2.8. Determination of Total Ash

Total ash was determined by ashing the sample at 550°C using a muffle furnace. Porcelain dishes were placed in a muffle furnace (Carbolite, Aston Lane, Hope, Sheffield, England, UK) for 30 min at 550°C. The dishes were cooled in desiccators (with granular silica gel) for about 30 minutes and weighed to the nearest milligram (*W*_1_). About three grams of fresh sample in duplicate was placed in dishes (*W*_2_). The dishes were placed on a hot plate under a fume hood, the temperature was slowly increased until smoking ceases, and the samples become thoroughly charred. The dish with sample was placed inside the muffle furnace at 550°C for an hour and cooled in desiccators for 1 hour. The crucible was removed from the furnace and allowed to cool to room temperature. The cooled samples were moistened again with a few drops of deionized water and allowed to evaporate in a hot plate. Again, it was ashed at 550°C for 30 minutes and the crucible was allowed to cool; some drops of deionized water and 5 drops of concentrated HNO_3_ were added and evaporated. Again, the content was reached at 550°C for 30 minutes. When cooled to room temperature, each dish with ash was reweighed to the nearest milligram (*W*_3_). Then, the total ash was determined as follows:

(4) $$\begin{array}{c} \text{Ash}\left(\%\right)=\frac{\left({\text{W}}_{3}-{\text{W}}_{1}\right)*100}{{\text{W}}_{2}-{\text{W}}_{1}}, \end{array}$$

where *W*_1_ is the weight of dried dish, *W*_2_ is the weight of fresh sample and dried dish, and *W*_3_ is the weight of dish and ash.

### 2.9. Determination of Total Carbohydrate

The total carbohydrate content was determined by difference (subtracting the moisture content, crude protein, total ash, and fat from the total dry weight of the sample).

(5) $$\begin{array}{c} \text{Carbohydrate }\left(\%\right)=100-\left(\text{fat}\right.\;\left(\%\right)+\text{protein}\left(\%\right)+\text{ash}\left(\%\right)+\text{moisture}\left(\%\right). \end{array}$$

### 2.10. Determination of Total Gross Energy

Gross energy was determined by summing energy from fat, carbohydrate, and protein contents using Atwater's conversion factors considering protein and carbohydrate each gives 4 kcal and fat yields 9 kcal per 100 gram [26].

(6) $$\begin{array}{c} \text{Gross energy }\left(\text{Kcal}/100\text{ g}\right)=9*\text{fat}+4*\text{protein}+4*\text{carbohydrate}. \end{array}$$

### 2.11. Determination of Total Minerals (Zinc, Iron, and Calcium)

Minerals were determined using the Shimadzu atomic absorption spectrophotometer (Model: AAS6800). Crucibles were washed with 6 N HCl and glasswares with 10% nitric acid. Then, the crucibles were placed in an oven for 30 minutes at 100°C and cooled in desiccators for 30 minutes. Then, 2.5 g of sample was weighed and charred at a hot plate starting from low temperature under a hood. Dry ashing, step 1: ashes were obtained by ashing samples in a muffle furnace at 550°C for 1 hour. The crucible was cooled and moistened with a few drops of deionized water, and water was evaporated on a hot plate. Again, the sample was ashed for 30 minutes at 550°C and cooled; a few drops of deionized water and 5 drops of concentrated HNO_3_ were added and allowed to evaporate on a hot plate. Finally, the sample was ashed once again for 30 minutes at 550°C, the crucible was cooled in desiccators for 1 hour, and the content was weighed. Dissolution, step 2: the ash was treated with 5 ml of 6 N HCl to wet it completely and dried on a low temperature hot plate. A 7 ml of 3 N HCl was added to the dried ash and heated on the hot plate until the solution just boils. The ash solution was cooled to room temperature and filtered through a filter paper (Whatman 42, 125 mm) into graduated flask. A 5 ml of 3 N HCl was added into each crucible dishes and heated until the solution just boiled, cooled, and filtered into the flask. The crucible dishes were again washed three times with deionized water; the washings were filtered into the flask. A 2.5 ml of 10% lanthanum chloride solution was added into each graduated flask. Then, the solution was cooled and diluted to the mark (50 ml) with deionized water. A blank was prepared by using the same procedure as the sample.

(7) $$\begin{array}{c} \text{Minerals }\left(\text{iron},\text{zinc},\text{and calcium}\right)\left(\text{mg}/100\text{ g}\right)=\frac{\left[\left(a-b\right)*V\right]}{10*W}, \end{array}$$

where *W* is the weight (g) of samples; *V* the volume (V) of extract; *a* is the concentration (*μ*g/ml) of sample solution; *b* is the concentration (*μ*g/ml) of blank solution.

### 2.12. Determination of Total Phytate

Phytate was determined by the method of Latta and Eskin (1980) and later modified by Vantraub and Lapteva [27]. About 0.1000 g of fresh samples was extracted with 10 ml 2.4% HCl in a mechanical shaker (Eberbach) for 1 hour at an ambient temperature and centrifuged at 3000 rpm for 30 minutes. The clear supernatant was used for phytate estimation. A 2 ml of Wade reagent (containing 0.03% solution of FeCl_3_·6H_2_O and 0.3% of sulfosalicylic acid in water) was added to 3 ml of the sample solution (supernatant), and the mixture was mixed on a Vortex (Maxi Maxi II) for 5 seconds. The absorbance of the sample solutions was measured at 500 nm using the UV-VIS spectrophotometer (Beckman DU-64-spectrophotometer, USA). A series of standard solutions were prepared containing 0, 5, 10, 20, and 40 *μ*g/ml of phytic acid (analytical grade sodium phytate) in 0.2 N HCl. A 3 ml of standard was added into 15 ml of centrifuge tubes with 3 ml of water which were used as a blank. A 1 ml of the Wade reagent was added to each test tube, and the solution was mixed on a Vortex mixer for 5 seconds. The mixtures were centrifuged for 10 minutes, and the absorbance of the solutions (both the sample and standard) was measured at 500 nm by using deionized water as a blank. A standard curve was made from absorbance versus concentration, and the slope and intercept were used for calculation.

(8) $$\begin{array}{c} \text{Phytate }\left(\text{mg}/100\text{ g}\right)=\frac{\left(\text{Absorbance}-\text{Intercept}\right)*3}{\text{Slope}*\rho *\text{weight of sample}*10}, \end{array}$$

where *ρ* is density.

### 2.13. Determination of Phytic Acid to Mineral Ratio

The mole of phytate and minerals (zinc, iron, and calcium) was determined by dividing the weight of phytate and minerals with its atomic weight. The molar ratio between phytate and zinc was obtained after dividing the mole of phytate with the mole of minerals. Thus, the phytate zinc molar ratio was calculated using the following formula [28]:

(9) $$\begin{array}{c} \text{Phytate to mineral ratio}=\frac{\text{phytic acid }\left(\text{mg}/100\text{ g}\right)/660}{\text{minerals }\left(\text{mg}/100\text{ g}\right)/\text{atomic weight}}. \end{array}$$

### 2.14. Sensory Acceptability Tests

Flatbread transferred into identical plates coded with three-digit random numbers. Triplicate samples from the same treatments were served for the acceptability test. Taste, color, flavor, mouth fullness, appearance, and overall acceptance of flatbread samples were rated by 15 untrained panelists using a 9-point hedonic scale (9 = like extremely and 1 = dislike extremely) [29, 30]. Finally, the rated sensory acceptability test scores were averaged.

### 2.15. Experimental Design and Statistical Analysis

Proximate composition, minerals, and vitamin C data were entered into a Statistical Package for the Social Sciences (SPSS) version 20 software program [31]. A completely randomized design (CRD) was used for nutritional composition data analysis. Randomized complete block design (RCBD) was used for sensory acceptability test to block error from between and within treatment and panelists. The significant mean differences of nutritional composition and sensory acceptability among groups were tested by one-way analysis of variance (ANOVA) and two-way ANOVA, respectively. Mean separation was done using the Tukey significance difference test at a *p* value less than 0.05. Finally, the result was described by means and standard deviation.

## 3. Result

### 3.1. Proximate Composition of Flatbread

The Proximate composition of the flatbread formulated from maize flour with diffrent levels of stinging nettle (*Urtica simensis*) is indicated in Table 2. The moisture content of the SN0 (control group) was significantly (*p* < 0.05) different from the treatment groups. As the proportion of nettle leaf flour increased, the moisture content slightly decreased from 10.68 in control to 8.44% in SN25 (25% nettle leaf flour supplemented) flatbread. The crude protein content of the SN0 was not significantly different from SN10 (10% supplemented bread), but it was different from SN15 (15% nettle supplemented), SN20 (20% nettle supplemented), and SN25. The value of crude protein showed a slight decrement with increasing the proportion of nettle flour supplement. The crude fat and total ash of the control group did not show a significant difference (*p* > 0.05) from SN10 and SN15, but it was significantly (*p* < 0.05) different from SN20 and SN25. Crude fat content decreased, and the total ash increased when the proportion of nettle flour supplement increased. Except for SN10, which did not show a difference, the crude fiber content of the control group (SN0) was significantly different from SN15, SN20, and SN25. The total carbohydrate value of SN0 was significantly different from all nettle flour-supplemented flatbread. Both crude fiber and total carbohydrate contents slightly increased with increasing the amount of nettle flour. The gross energy value of SN0 was not significantly (*p* > 0.05) different from nettle flour-supplemented flatbread.

### 3.2. Selected Minerals, Vitamin C Content, Phytic Acid, and Zinc to Phytate Molar Ratio

Table 3 depicts the values of selected minerals, vitamin C, phytic acid, and zinc to phytate molar ratio. The minerals (calcium, iron, and zinc) and vitamin C content showed a significant (*p* < 0.05) increment when the amount of nettle flour added increased. The calcium, iron, and zinc content increased by approximately 5 folds, 2 folds, and 2 folds in 25% nettle flour-supplemented flatbread compared to the control group, respectively. Vitamin C was not detected in the control, but 12.61 *μ*g, 23.99 *μ*g, 34.99 *μ*g, and 47.57 *μ*g in 10%, 15%, 20%, and 25% nettle flour supplemented flatbread, respectively. Regarding phytate, the numeric value of phytate slightly decreased (84.64-70.47 mg/100 g) when comparing the control and supplemented groups. However, the content showed increment within the treatment group. There was a significant difference (*p* < 0.05) in the phytate content of the control and treatment groups. Similarly, the phytate to the mineral molar ratio (phytate : zinc, phytate : iron, and phytate : calcium) was decreased when the proportion of nettle flour increased.

### 3.3. Sensory Acceptability

The result of sensory acceptability of the nettle flour-supplemented unleavened maize flatbread is depicted in Table 4. The mean and standard deviation of all sensory quality parameters decreased with increasing the proportion of nettle flour. The control sample had the highest rating, and the treatment group rated from liked moderately to liked slightly except for the color which was neither liked nor disliked. There is a highly statistically significant difference (*p* < 0.05) between the color preference of control and the other four treatments. The taste and flavor of SN15 and SN20, the appearance of SN10, and the mouthfeel of SN10 and SN15 were not significantly (*p* > 0.05) different from the control group. All the nettle leaf flour-supplemented flatbread was rated like very much at 10% supplementation.

## 4. Discussion

The result of proximate composition indicated that the moisture and crude protein contents of flatbread were slightly decreased with an increase in the proportion of nettle leaf flour. A similar finding was reported in pasta made from wheat, oat, and moringa leaf flour [32]. However, contradicting result was reported in noodles [33] and bread [34] made from wheat and nettle leaf flour. The finding is also contradicting the result reported for flatbread made from maize (Zea mays L.) and orange-fleshed sweet potato (Ipomoea batatas L.) flour [4], in which moisture content significantly increased with the increasing proportion of nettle flour. The moisture content of a particular product determines the microbial growth and shelf life of the product [35]. The decreased moisture content with increasing nettle flour might give longer shelf life compared to the control group.

The crude protein and fat contents were significantly decreased from 11.2% to 7.21% and 1.12 to 0.48%, respectively, with an increase in the amount of nettle flour from 0% to 25%. The finding is similar to another study in Ethiopia [4]. The increased crude fat with an increase in the level of nettle flour in this study is also inline, yet crude protein contradicts with another study [33]. The finding also disagrees with other studies [29, 32]. The decreased fat and protein contents in this study might be explained by the low content of these nutrients in nettle leaf. The difference may be due to differences in recipes such that maize in this study and wheat in compared studies were used as the main recipe. The difference might also be due to variety, genotype, climate, soil, vegetative stage, harvest time, storage, processing, and treatment since these variables could affect the chemical composition of nettle [36, 37]. The reduced fat content may be important to limit unnecessary dietary fat intake.

The total carbohydrate content was increased from 75.33% to 80.05% in the highest supplemented (SN25) flatbread. Contradicting result was recorded in noodles from wheat and nettle leaf flour [33], pasta from moringa leaf, oat, and wheat [32] in which carbohydrate content was decreased with increasing nettle leaf flour and moringa leaf flour, respectively. The present finding also disagrees with another study in which carbohydrate content did not show significant change with increasing nettle leaf flour in bread from wheat and nettle leaf flour [34]. The increase in total carbohydrate in this study might be due to the high fiber content of nettle leaf and raw material difference. The crude fiber content increased from 2.14% in control to 3.05% in the highest supplemented flatbread. A similar result was reported in other studies [29, 30, 33].

The gross energy content of the control group and nettle-supplemented flatbread was not significantly different. This might be due to a decrease in crude protein and fat content and increased total carbohydrate with increasing nettle flour content. Thus, the reduced energy value due to decreased protein and fat content is likely compensated by increased carbohydrate content since the gross energy was coming from the three macronutrients. The result disagrees with the finding by [33], in which the gross energy was decreased (367.38 to 351.43 Kcal/100 g) with increasing nettle leaf flour, but in line with finding by [34], in which gross energy was increased from 223.56 to 232.72 Kcal/100 g with increasing nettle flour leaf. A similar result was reported in pasta from oat, wheat, and moringa leaf flour where the gross energy increased from 357.00 in control to 370.64 Kcal/100 g by 25% moringa leaf incorporation [32].

According to the present study, the total ash content was increased from 1.84% in control to 3.81% in 25% nettle flour-supplemented flatbread. Comparable results were recorded in other studies [29, 30, 33]. The high ash content of the product is indicative of the presence of high mineral contents. Similarly, the increasing total ash content with increasing nettle flour reflects the dried nettle leaf flour has a high amount of minerals than maize. There was a significant increment in the mineral content of the blend. The calcium, iron, and zinc content of nettle leaf-incorporated flatbread was nearly 5 folds, 2 folds, and 2 folds higher compared to the control group. This finding is in line with a study from Ethiopia such that calcium, zinc, and mineral content were significantly increased with the increased proportion of nettle flour in wheat flour-based nodules [33]. The result is in agreement with other findings where mineral (calcium, zinc, iron, and phosphorous) content of cookies developed from wheat and moringa leaf significantly increased with increasing ratios of moringa leaf [38].

The phytic acid content of flatbread was reduced by 16.7-20.2% with the increasing proportion of nettle leaf flour from 10% to 25%. However, the content showed increment within the supplemented group. The decreased in phytate content when increasing nettle flour might be due to the low proportion of maize and activation of phytase enzyme during germination. On the other hand, the increased phytate contents within nettle leaf-supplemented flatbread may be due to increased nettle leaf flour. This might need further processing such as blanching or soaking or searching for another drying method that minimizes antinutritional factor (phytate) without compromising the nutritional composition of nettle leaf. According to the international zinc consultative group suggestion, the phytate to zinc molar ratio is an important index for zinc bioavailability, and a ratio of less than 15 indicates bioavailability. A ratio of less than five has good bioavailability [28]. In the present study, the phytic acid to zinc molar ratio was decreased from 7.49 in control to 2.79 in the highest supplemented flatbread. Similarly, the phytate to iron molar ratio and phytate to calcium molar ratio significantly decreased when the proportion of nettle flour supplements was increased. This suggests that phytate : zinc, phytate : iron, and phytate : calcium molar ratio of all nettle-supplemented flatbread was below a threshold that estimates a good bioavailability of the respective minerals. The low phytate to selected mineral ratio implies bioavailability of the studied minerals (calcium, iron, and zinc) which may not be affected by the presence of phytate.

Sensory qualities such as taste, color, odor flavor, and texture are the most important feature for the formulation of a new product than quantity [39]. Thus, it is strongly believed that evaluating the sensory acceptability test of a new product is an inevitable activity besides looking at the nutritional, safety, and convenience of a given product [33]. The present study indicated that all sensory acceptability tests were decreased when increasing the proportion of nettle leaf flour. The color acceptability of flatbread was significantly (*p* < 0.05) reduced with increasing the proportion of nettle leaf flour. The result is in agreement with other studies [29, 30, 38]. In addition to the natural color of nettle itself, research evidence recognized that drying affects sensory parameters like aroma, flavor, and appearance [40]. Drying changes the aroma of food products through losses in volatile compounds or the formation of new volatile compounds as a result of oxidation and esterification reactions [41, 42]. Hence, the reduced sensory acceptability could be explained by the change of flavor, aroma, and appearance of nettle leaf during drying. The color acceptability of the flatbread was decreased from 8.71 (liked very much) in a control group to 5.61 (neither liked nor disliked) in the highest supplemented (SN25) flatbread. Scholars illustrated that the loss of green color during drying is mainly due to the degradation of chlorophyll a and b, and carotenoid losses due to oxidation of the highly unsaturated carotenoid structure and cis-trans-isomerization during thermal processing. Hence, the color acceptability affected might be due to loss of green color by the degradation of chlorophyll and loss of carotenoids [41, 42]. The result of this finding was in agreement with [33], but contradicting with other studies [29, 32].

## 5. Limitation of the Study

This study did not address other antinutritional factors such as polyphenols that can affect the bioavailability of the minerals.

## 6. Conclusion

The crude protein and fat contents were decreased from 11.02 to 7.21 g and 1.12 to 0.48 mg when the nettle flour supplementation increased from 0% to 25%. The reduced fat content could be beneficial to limit unnecessary excess energy intake from diet and might contribute to the management of chronic diseases like obesity and overweight. On the other hand, the crude ash was increased from 1.84 to 3.81 g; the calcium, zinc, and iron content increased from 60.51 to 283.74, 1.72 to 3.94 mg, and 5.09 to 9.24 mg while the proportion of nettle flour supplementation increased from 0 to 25%. There may also decrease mineral deficiencies and contribute to healthy life. Vitamin C content was very low. On the other hand, increasing nettle flour decreased crude protein and crude fat content but has no effect on gross energy. Incorporating the nettle leaf flour with maize flour compromised and reduced the sensory acceptability of flatbread.

## 7. Recommendation

Optimum baking temperature and time combination that minimizes nutrient losses should be studied. Since the sensory acceptability is compromised with the increasing amount of nettle flour, the supplementary ratio of nettle flour should be reduced to a maximum of 15%. Treatments like soaking of nettle leaves with ethanol shock are recommended to increase recovery of nutrients as suggested by Maimaiti et al. [43]. On the other hand, techniques that improve organoleptic quality and sensory acceptability of stinging nettle leaf should be studied.

## Figures and Tables

**Table 1:** Treatment blending ratio of nongerminated maize (NGM) flour, germinated maize (GM) flour, and nettle (*Urtica simensis*) flour.

Treatments	Blending ratio		
	Nongerminated maize flour (NGMF)	Germinated maize flour (GMF)	Stinging nettle (*Urtica Simensis*) flour (SN)
SN0	100%	—	—
SN10	—	90%	10%
SN15	—	85%	15%
SN20	—	80%	20%
SN25		75%	25%

SN0 = 100% nongerminated maize flour (control); SN10 = 90% germinated maize flour + 10% nettle flour; SN15 = 85% germinated maize flour + 15% nettle flour; SN20 = 80% germinated maize flour + 20% nettle flour; SN25 = 75% germinated maize flour + 25% nettle flour.

**Table 2:** Means^1,2^ of the proximate composition (per 100 g) of flatbread formulated from maize with different levels of stinging nettle (*Urtica simensis*) flour.

Treatment	Blending ration			Moisture (%)	Protein (g)	Fat (g)	Ash (g)	Fiber (g)	CHO (g)	Gross energy (Cal)
	NGMF	GMF (%)	SN (%)							
SN0	100	—	—	10.68 ± 0.47^a^	11.02 ± 0.22^a^	1.12 ± 0.11^a^	1.84 ± 0.07^a^	2.19 ± 0.14^c^	75.33 ± 0.77^c^	355.52 ± 0.24^ab^
SN10	—	90	10	8.98 ± 0.11^b^	10.54 ± 0.49^ab^	0.97 ± 0.03^a^	2.24 + 0.16^a^	2.47 ± 0.08^bc^	77.27 ± 0.74^b^	359.97 ± 1.17^ab^
SN15	—	85	15	8.84 ± 0.13^b^	10.00 ± 0.32^b^	0.74 ± 0.11^b^	2.88 ± 0.07^b^	2.71 ± 0.1^ab^	77.53 ± 0.29^b^	356.81 ± 0.96^ab^
SN20	—	80	20	8.23 ± 0.27^b^	9.17 ± 0.20^c^	0.68 ± 0.05^b^	3.18 ± 0.07^b^	2.86 ± 0.08^a^	78.72 ± 0.56^ab^	357.76 ± 1.03^ab^
SN25	—	75	25	8.44 ± 0.45^b^	7.21 ± 0.12^d^	0.48 + 0.03^c^	3.81 + 0.34^c^	3.05 ± 0.24^a^	80.05 ± 0.66^a^	353.39 ± 3.31^b^

NGMF = nongerminated maize flour; GMF = germinated maize flour; SN = stinging nettle flour; SN0 = NGMF without nettle flour; SN10 = 90% GMF + 10% SN; SN15 = 85% GMF + 15% SN; SN20 = 80% GMF + 20% SN, SN25 = 75% GMF + 25% SN; 1 = values are averages of duplicate measurements (mean ± standard deviation); 2 = means with the same superscripts within the same column are not statistically significantly different from each other (*p* < 0.05); % = percent per 100 g; g = gram; cal = calorie.

**Table 3:** Means^1,2^ of selected minerals, vitamin C contents, and phytate to mineral molar ratio of flatbread formulated from maize with different levels of stinging nettle (*Urtica simensis*) flour.

Treatment	Blending proportions			Calcium (mg)	Iron (mg)	Zinc (mg)	Vitamin C (*μ*g)	Phytic acid (mg)	Phytate : Zn	Phytate : Fe	Phytate : Ca
	NGMF (%)	GMF (%)	SN (%)								
SN0	100	—	—	60.51 ± 0.44^e^	5.09 ± 0.12^d^	1.72 ± 0.12*c*	0.00 ± 0.00^e^	84.64 ± 0.08^a^	7.49 ± 0.49^a^	1.41 ± 0.03^a^	0.08 ± 0.00^a^
SN10	—	90	10	113.78 ± 4.25^d^	6.85 ± 0.57^c^	2.03 ± 0.18^c^	12.61 ± 0.09^d^	67.53 ± 1.08^b^	5.07 ± 0.51^b^	0.84 ± 0.08^b^	0.04 ± 0.00^b^
SN15	—	85	15	179.87 ± 0.12^c^	6.98 ± 0.01^c^	2.24 ± 0.22^c^	23.99 ± 0.02^c^	67.27 ± 0.81^b^	4.58 ± 0.47^b^	0.82 ± 0.01^b^	0.02 ± 0.00^c^
SN20	—	80	20	209.81 ± 10.87^b^	8.28 ± 0.30^b^	2.88 ± 0.06^b^	34.99 ± 0.10^b^	66.93 ± 0.79^b^	3.51 ± 0.04^c^	0.69 ± 0.02^c^	0.02 ± 0.00^d^
SN25	—	75	25	283.74 ± 0.37^a^	9.24 ± 0.27^a^	3.59 ± 0.32^a^	47.57 ± 2.67^a^	70.47 ± 1.04^b^	2.79 ± 0.54^c^	0.61 ± 0.02^c^	0.01 ± 0.00^e^

NGMF = nongerminated maize flour; GMF = germinated maize flour; SN = stinging nettle flour; SN0 = NGMF without nettle flour; SN10 = 90% GMF + 10% SN; SN15 = 85% GMF + 15% SN; SN20 = 80% GMF + 20% SN; SN25 = 75% GMF + 25% SN; 1 = values are averages of duplicate measurements (mean ± standard deviation); 2 = means with the same superscripts within the same column are not statistically significantly different from each other (*p* < 0.05).

**Table 4:** Means^1,2^ of sensory acceptability scores of the flatbread developed from stinging nettle and maize flour performed at laboratory level.

Treatment	Blending ratio			Sensory attributes					
	NGMF	GMF	SN	Color	Taste	Flavor	Appearance	Mouthfeel	Overall acceptability
SN0	100	—	—	8.71 ± 0.25^a^	8.22 ± 0.23^a^	8.71 ± 0.26^a^	8.73 ± 0.23^a^	8.58 ± 0.10^a^	8.59 ± 0.02^a^
SN10	—	90	10	7.97 ± 0.15^b^	7.47 ± 0.09^b^	7.80 ± 0.20^b^	8.26 ± 0.11^ab^	8.11 ± 0.12^a^	7.92 ± 0.09^b^
SN15	—	85	15	6.37 ± 0.19^c^	7.26 ± 0.17^bc^	7.56 ± 0.18^bc^	7.86 ± 0.28^bc^	7.89 ± 0.22^a^	7.59 ± 0.10^c^
SN20	—	80	20	6.92 ± 0.08^cd^	6.93 ± 0.25^cd^	7.21 ± 0.19^cd^	7.31 ± 0.09^cd^	6.77 ± 0.54^b^	7.03 ± 0.09^d^
SN25	—	75	25	5.61 ± 0.11^d^	6.45 ± 0.20^d^	6.73 ± 0.21^d^	6.87 ± 0.26^d^	6.57 ± 0.50^b^	6.65 ± 0.02^e^

NGMF = nongerminated maize flour; GMF = germinated maize flour; SN = stinging nettle flour; SN0 = NGMF without nettle flour; SN10 = 90% GMF + 10% SN; SN15 = 85% GMF + 15% SN; SN20 = 80% GMF + 20% SN, SN25 = 75% GMF + 25% SN; 1 = values are averages of triplicate measurements (mean ± standard deviation) of 15 untrained panelists at laboratory level; 2 = means with the same superscripts within the same column are not statistically significantly different from each other (*p* < 0.05).

## Data Availability

Data used for this research and analysis is available from the corresponding author and will be provided upon reasonable request.
